# Fecal prevalence, serotype distribution and antimicrobial resistance of *Salmonellae* in dairy cattle in central Ethiopia

**DOI:** 10.1186/s12866-016-0638-2

**Published:** 2016-02-16

**Authors:** Tadesse Eguale, Ephrem Engidawork, Wondwossen A. Gebreyes, Daniel Asrat, Haile Alemayehu, Girmay Medhin, Roger P. Johnson, John S. Gunn

**Affiliations:** Aklilu Lemma Institute of Pathobiology, Addis Ababa University, P.O. Box 1176, Addis Ababa, Ethiopia; Department of Pharmacology and Clinical Pharmacy, School of Pharmacy, College of Health Sciences, Addis Ababa University, Churchill Avenue, P.O. Box 1176, Addis Ababa, Ethiopia; Department of Veterinary Preventive Medicine, The Ohio State University, 1920 Coffey Rd., Columbus, OH 43210 USA; Department of Microbiology, Immunology & Parasitology, School of Medicine, College of Health Sciences, Addis Ababa University, Churchill Avenue, P.O. Box 9086 Addis Ababa, Ethiopia; Laboratory for Foodborne Zoonoses, 110 Stone Road West, Guelph, ON N1G 3W4 Canada; Department of Microbial Infection and Immunity, Center for Microbial Interface Biology, The Ohio State University, Biomedical Research Tower, 460 West 12th, Columbus, OH 43210-1214 USA

**Keywords:** Dairy farm, Feces, *Salmonella enterica*, Multi-drug resistance

## Abstract

**Background:**

*Salmonellae* are major worldwide zoonotic pathogens infecting a wide range of vertebrate species including humans. Consumption of contaminated dairy products and contact with dairy cattle represent a common source of non-typhoidal *Salmonella* infection in humans. Despite a large number of small-scale dairy farms in Addis Ababa and its surrounding districts, little is known about the status of *Salmonella* in these farms.

**Results:**

*Salmonella* was recovered from the feces of at least one animal in 7.6 % (10/132) of the dairy farms. Out of 1203 fecal samples examined, 30 were positive for *Salmonella* resulting in a weighted animal level prevalence of 2.3 %. Detection of diarrhea in an animal and in a farm was significantly associated with animal level (*p* = 0.012) and herd level (*p* < 0.001) prevalence of *Salmonella.* Animal level prevalence of *Salmonella* was significantly associated with age (*p* = 0.023) and study location; it was highest among those under 6 months of age and in farms from Adaa district and Addis Ababa (*p* < 0.001). Nine different serotypes were identified using standard serological agglutination tests. The most frequently recovered serotypes were *Salmonella* Typhimurium (23.3 %), *S*. Saintpaul (20 %), *S*. Kentucky (16.7 %) and *S*. Virchow (16.7 %). All isolates were resistant or intermediately resistant to at least one of the 18 drugs tested. Twenty-six (86.7 %), 19 (63.3 %), 18 (60 %), 16 (53.3 %) of the isolates were resistant to streptomycin, nitrofurantoin, sulfisoxazole and tetracycline , respectively. Resistance to 2 drugs was detected in 27 (90 %) of the isolates. Resistance to 3 or more drugs was detected in 21 (70 %) of the isolates, while resistance to 7 or more drugs was detected in 11 (36.7 %) of the isolates. The rate of occurrence of multi-drug resistance (MDR) in *Salmonella* strains isolated from dairy farms in Addis Ababa was significantly higher than those isolated from farms outside of Addis Ababa (*p* = 0.009). MDR was more common in *S.* Kentucky, *S.* Virchow and *S.* Saintpaul.

**Conclusion:**

Isolation of *Salmonella* serotypes commonly known for causing human salmonellosis that are associated with an MDR phenotype in dairy farms in close proximity with human population is a major public health concern. These findings imply the need for a strict pathogen reduction strategy.

## Background

*Salmonella* is a diverse bacterial species comprising over 2600 serotypes [1]. *Salmonella* commonly colonizes a range of animal hosts such as mammals, amphibians, reptiles, birds and insects [2]. There are 2 species of *Salmonella*: *Salmonella enterica* and *Salmonella bongori. Salmonella enterica* is further classified into 6 subspecies (*Salmonella enterica* subspecies *enterica*, *S. enterica* Subspecies *salmae*, *S. enterica* Subspecies *arizonae*, *S. enterica* Subspecies *diarizonae*, *S. enterica* Subspecies *hautenae* and *S. enterica* Subspecies *indica*). Most of the *Salmonella* serotypes are part of *S. enterica* subspecies *enterica*, and over 99 % of human and animal infections are caused by serotypes under this subspecies [3].

Diseases caused by *Salmonella* represent an important public health problem among the common bacterial foodborne pathogens worldwide. It is estimated that globally 93.8 million cases and 155,000 deaths are associated with gastroenteritis due to *Salmonella* species annually. Of these cases, 85.6 % were estimated to be foodborne [4]. Human salmonellosis has been associated with contaminated food products, mainly those of animal origin such as poultry, beef, pork and dairy products, as well as direct contact with infected animals [5–7].

Various serotypes of *Salmonella* have been isolated from the feces of apparently healthy dairy cattle. *Salmonella* in dairy animals may exist as a normal microbiota of the gastrointestinal population, or as a transient member of the gastrointestinal microbial population [8].

All sick, recovered and asymptomatic cattle can shed *Salmonella* through feces and the organism can survive for a long time in favorable environments outside the host [9]. Fecal shedding of *Salmonella* can increase intra-herd transmission, accidental spread to other herds, environmental contamination and risk of human infection [10]. Consumption of raw milk, inadequately pasteurized milk, improperly cooked beef from culled dairy cattle, contaminated water and direct animal contact are the major routes of acquiring dairy associated salmonellosis in humans [6].

In Ethiopia, there are large numbers of small-scale peri-urban dairy farms mainly situated close to areas of public residence. Most of these farms are located very close to Addis Ababa, capital city of the country, or reside within the city in a very close proximity with human populations. The consumption of raw milk and its derivatives is common in Ethiopia, posing high risk of infection with dairy-associated foodborne pathogens. Such pathogens include *Salmonella spp., Klebsiella* spp., *Enterobacter* spp., and *Escherichia coli*, which have been identified in milk products in Ethiopia [11]. Gram positive pathogens such as *Staphylococcus aureus, Bacillus cereus, Listeria monocytogenes* and *Enterococcus* spp. have also been frequently isolated from milk [12, 13].

Occurrence of non-typhoidal *Salmonella* serotypes commonly infecting humans in dairy cattle, particularly, those stains resistant to antimicrobial agents commonly used in human medicine, are a serious threat to human health. Some multi-drug resistant (MDR) *Salmonella* outbreaks in humans have been linked to exposure to dairy farms or contaminated dairy products [6, 14]. Information on the prevalence, serotype distribution and antimicrobial susceptibility of *Salmonella* in dairy farms is vital to implementation of appropriate strategies to prevent the introduction and spread of the pathogen in the farm as well as to reduce the risk of human salmonellosis. Knowledge on the serotypes circulating in dairy farms would inform scientists/clinicians on the role of dairy cattle as a source of human *Salmonella* infections. A previous study conducted in Addis Ababa has shown farm level prevalence of 47.8 % and animal level prevalence of 7.7 % [15]. However, this study involved small sample size and the isolates were not serotyped. Given the relative lack of information concerning the prevalence and serotype distribution of *Salmonella* spp. in dairy farms in Ethiopia, the present study was designed to investigate animal level and herd level fecal prevalence of *Salmonella*, serotype distribution and antimicrobial resistance profiles of *Salmonella* in dairy farms in and around Addis Ababa, Ethiopia. It also attempted to investigate the association of farm size, occurrence of diarrhea in the farm and age of animals with prevalence of *Salmonella* in these dairy farms.

## Methods

### Study design, study area and sampling of study animals

**Fig. 1:**
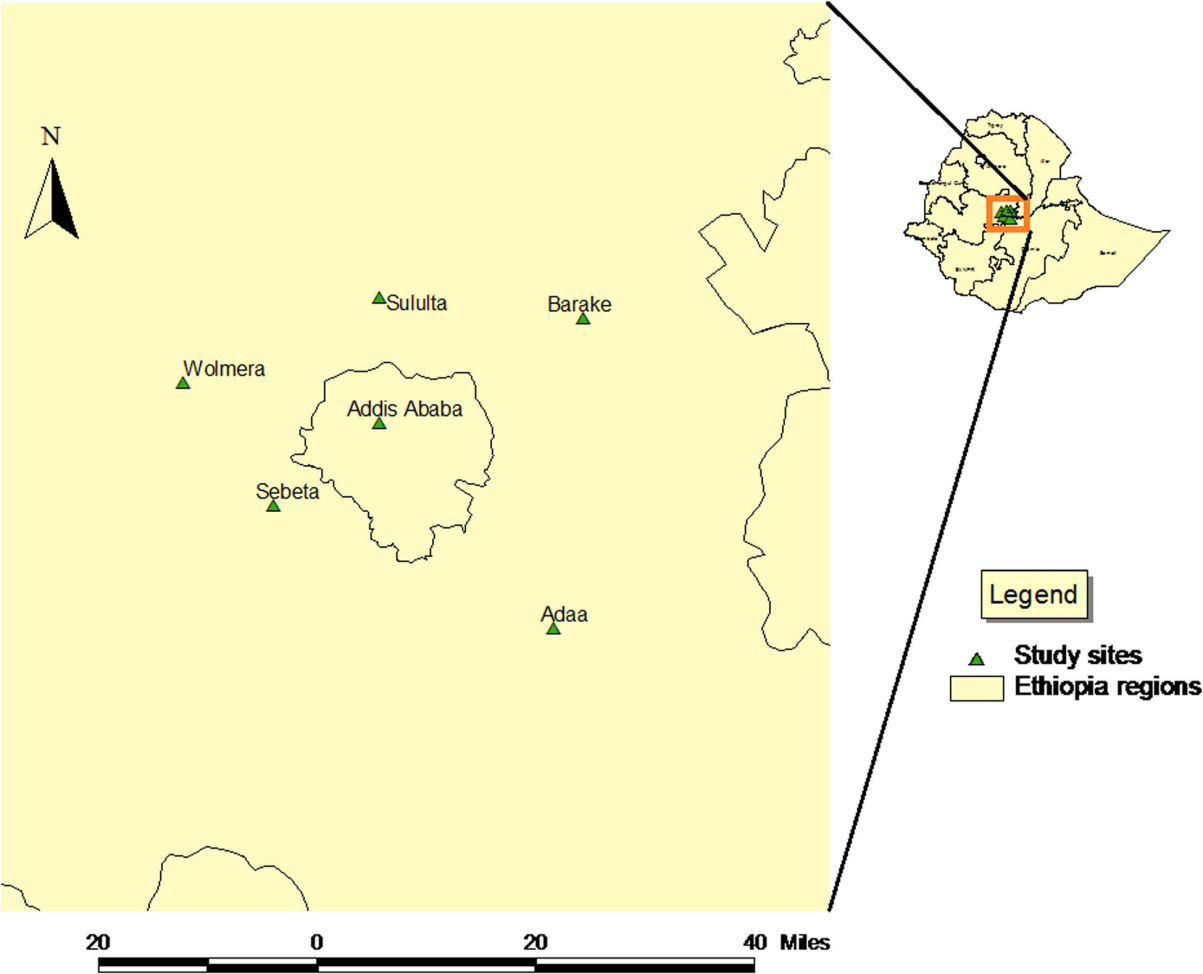
Locations of Addis Ababa and surrounding districts where fecal samples from dairy cattle were collected (source: Original Ethiopian shape file was obtained from Ethiopian Mapping Agency)

A cross-sectional study was conducted in Addis Ababa and in five districts of the Oromia region located at the outskirt of Addis Ababa, namely: Sebeta, Barake, Welmera, Sululta and Adaa (Fig. 1). In these areas, the interaction between animal and human population is very high due to high density of the human populations and the large number of peri-urban dairy farming facilities. These areas are the major sources of dairy milk supply to Addis Ababa. Sampling of study herds and animals was conducted from June to December 2013. Study animals were selected from 132 dairy herds (Addis Ababa; *n* = 38; Adaa, *n* = 12; Sebeta, *n* = 21; Sululta, *n* = 24; Welmera; *n* = 18). Inclusion of herd in the sampling was based on representation of the area under study, willingness of the owners, geographical accessibility, and the herd having a minimum of 5 cattle. The largest herd size contained 398 head of cattle. Farms were categorized into small (5–20 animals in a herd), medium (21–50 animals in a herd) and large (more than 50 animals). Mean herd size of small, medium and large farms was 12.6, 31.7 and 100.4, respectively. In total 1203 fecal samples were collected from healthy as well as diarrheic cattle during the study period. The study design was cross-sectional implying a one point fecal sample collection from a given herd and hence there was no repeated fecal sample collection.

### Sample collection, *Salmonella* isolation and identification

Fecal samples were collected directly from the rectum using disposable gloves into sterile zippered plastic bags and transported to the Microbiology Laboratory, Aklilu Lemma Institute of Pathobiology, in an ice box within 3–4 h of collection. Isolation and identification of *Salmonella* was conducted using conventional methods [16, 17]. Briefly, 10 g of feces was pre-enriched in 90 ml of buffered peptone water (BPW) (Becton Dickinson, Sparks, MD) and incubated overnight at 37 °C. A 100 μl pre-enriched suspension was added into 9.9 ml of Rappaport-Vassiliadis enrichment Broth (RVB) (Oxoid, USA) and incubated at 42 °C for 24 h. At the same time, 1 ml of suspension was also transferred to 10 ml of Tetrathionate broth (TTB) (Oxoid, USA) and incubated for 24 h at 37 °C. It was then streaked from both RVB and TTB to Xylose Lysine Tergitol 4 (XLT-4) (Oxoid, USA) selective media and the plates were incubated at 37 °C for 24 to 48 h. Presumptive *Salmonella* colonies were further investigated biochemically using Triple Sugar Iron agar, Urea, Citrate and Lysine Iron Agar slants. Those colonies with typical *Salmonella* biochemical properties were then further confirmed by genus specific PCR [18]. A reference strain of *S.* Typhimurium (ATCC 14028) was used as a positive control during biochemical analysis and PCR. One confirmed *Salmonella* isolate from each positive sample was stored at −80 °C in 20 % glycerol until further testing.

### Data collection

Information such as herd size, housing condition, types of antimicrobials commonly used in the farm, age, sex of animals and presence of diarrhea in a farm was recorded using a purposively designed questionnaire. A farm was categorized as a diarrheic farm if one or more animals in the herd were diarrheic at the time of sample collection. Collection of data was performed at the time of fecal sample collection from each farm.

### *Salmonella* serotyping and phage typing

*Salmonella* isolates were serotyped and phage typed at the World Organization for Animal Health (OIÉ) Reference Laboratory for Salmonellosis of the Public Health Agency of Canada’s National Microbiology at Guelph. Serovars were determined by serum agglutination according to the White-Kauffmann-Le Minor scheme [19, 20], with identification of somatic (O) antigens by slide agglutination tests [21] and flagellar (H) antigens by a microplate agglutination technique [22]. *S.* Typhimurium isolates were phage typed by the methods developed initially by Callow [23] and extended by Anderson et al. [24] and Rabsch [25] with 30 reference phages obtained directly from the WHO Reference Laboratory for phage typing of *Salmonella* species at Public Health England or from the same source via Canada’s National Microbiology Laboratory at Winnipeg. These typing phages were number 1–35 with discontinued use of phages 9, 30, 31, 33 and 34. Internal reference strains of phage type 1 (fully susceptible) and phage type 124 (susceptible to only one phage) were included as controls. Isolates that reacted with the phages but did not conform to any recognized phage type were designated atypical (AT), while those that did not react with any of the typing phages were designated untypeable (UT).

### Antimicrobial susceptibility testing

Susceptibility of the isolates to 18 antimicrobials was determined using the Kirby-Bauer disk diffusion method according to the guidelines of the Clinical and Laboratory Standards Institute [26]. The following antimicrobials (Sensi-Discs, Becton, Dickinson and Company, Loveton, USA) and disc potencies (μg) were used: amikacin (30), amoxicillin + clavulanic acid (20/10), ampicillin (10), cefoxitin (30), ceftriaxone (30), cephalothin (30), chloramphenicol (30), ciprofloxacin (5), gentamicin (10), kanamycin (30), nalidixic acid (30), neomycin (30), nitrofurantoin (100), streptomycin (10), sulfisoxazole (1000), sulfamethoxazole + trimethoprim (23.75/1.25), trimethoprim (5) and tetracycline (30). The interpretation of the categories of susceptible, intermediate or resistant was based on the CLSI guidelines [26]. For the purpose of analysis, all readings classified as intermediate were considered as resistant unless indicated.

*E. coli* ATCC 25922 was used as a quality control organism.

### Statistical analysis

The data analysis method that fits to survey data as it is implemented in STATA version 12 was used to estimate prevalence of *Salmonella* and to investigate its association with pre-specified background characteristics. In animal level analysis, the probability of selecting a given animal from a given herd was considered as a weighting variable. Animal level prevalence of *Salmonella* was calculated as the weighted percentage of *Salmonella* culture-positive fecal samples among the total number of animals examined. Herd level prevalence of *Salmonella* was calculated as the percentage of herds with one or more *Salmonella* culture-positive fecal samples among the total number of herds sampled. Association of weighted animal level prevalence and selected background characteristics was assessed using pearson chi-square within survey command of STATA software. Association of herd level *Salmonella* positivity and pre-specified characteristics was assessed using pearson chi-square. The difference between mean numbers of antimicrobials to which isolates were resistant was compared using a student *t*-test. Results were reported as being statistically significant whenever the *p*-value was less than 0.05.

### Ethics statement

Ethical clearance for the study was obtained from the National Research Ethics Review Committee, Ethiopia. Informed oral consent was obtained from the farm owners at the time of sample collection.

## Results

### Prevalence and risk factors

Weighted animal level *Salmonella* prevalence was 2.3 % and at least one *Salmonella* positive animal was detected in 7.6 % (10/132) of the herds examined. There was no significant difference in prevalence of *Salmonella* between male and female animals. Significant difference in the prevalence of *Salmonella* was observed across different age groups (*p* = 0.023) and the largest was observed in cattle less than 6 months old. Similarly, animal level prevalence of *Salmonella* among study sites was significantly different (*p* < 0.001): highest prevalence was 5 % in Adaa followed by 4.2 % in Addis Ababa and 2.0 % in Sebeta (Table 1). However, herd level prevalence of *Salmonella* was not significantly different among study sites (Table 2). Diarrhea was detected in 34 of 1203 animals. Three of the diarrheic animals were positive for *Salmonella* whereas 27 out of 1169 non-diarrheic animals were positive for *Salmonella.* These 3 *Salmonella* positive diarrheic animals were a 2 week old calf infected with *S*.Typhimurium var. Copenhagen on a farm in Adaa district, a 3 month old calf infected with *S*. kentucky in Addis Ababa and a 6 year old cow infected with *S*. Dublin in the Sebeta district. Detection of diarrhea in an animal was significantly associated with animal level *Salmonella* carriage (*p* = 0.012) (Table 1). Detection of diarrhea in one or more animals in a farm was also significantly associated with herd level prevalence of *Salmonella* (*p* < 0.001) (Table 2). Out of 255 fecal samples collected from 24 diarrheic herds, 22 were positive for *Salmonella* whereas, only 8 of the 948 fecal samples collected from 108 non-diarrheic herds were positive for *Salmonella*. Six of 24 (25 %) of diarrheic herds were positive for *Salmonella* while only 4 of 108 (3.7 %) of non-diarrheic herds were positive for *Salmonella* (*p* < 0.001).

**Table 1:** Animal level prevalence of *Salmonella* and its unadjusted association with selected characteristics

Characteristics	Categories	Number	Weighted ^a^percent positive for *Salmonella*	*p*-value
Sex	Male	101	1.4	0.483
	Female	1102	2.4	
Age	<6 month	280	4.5	0.023
	6 months–2 years	162	0.0	
	2 years–5 years	143	2.9	
	5 years–8 years	496	1.6	
	≥8 years	122	2.5	
Study site	Sebeta	141	2.0	<0.001
	Addis Ababa	319	4.2	
	Adaa	184	5.0	
	Barake	151	0.4	
	Welmera	195	0.0	
	Sululta	213	0.2	
Herd Size	Small [5–20)	480	1.8	0.117
	Medium [20–50)	369	2.1	
	Large [50+	354	4.3	
Have diarrhoea	No	1169	2.1	0.012
	Yes	34	9.4	
Overall		1203	2.3	

^a^The result was weighted by the probability of selecting animals from its respective farm

**Table 2:** Herd level prevalence of *Salmonella* and its unadjusted association with selected farm level characteristics

Characteristics	Categories	Number of farms studied	Percent positive for *Salmonella*	*p*-value
Study site	Sebeta	20	5.0	0.372
	Addis Ababa	38	13.2	
	Adaa	12	16.7	
	Barake	19	5.3	
	Welmera	18	0.0	
	Sululta	25	4.0	
Herd Size	Small [5–20)	79	3.8	0.047
	Medium [20–50)	33	9.1	
	Large [50+	20	20.0	
Farm diarrhoea status	Diarrheic	24	25.0	<0.001
	Non-diarrheic	108	3.7	
Overall		132	7.6	

There was no significant difference in animal level prevalence of *Salmonella* among animals from farms of different herd size (*p* = 0.117) (Table 1). However, herd level prevalence of *Salmonella* was significantly higher in farms with large herd size (*p* = 0.047) (Table 2). All 30 *Salmonella* isolates were obtained from herds that were kept completely indoors, while none was recovered from farms that allowed their animals to graze outside occasionally or those where cattle were totally outdoors.

### *Salmonella* serotype distribution

Nine different serotypes were identified (Table 3). *S*. Typhimurium, grouped with its variant *S.* Typhimurium var. Copenhagen, was the most common (7/30, 23.3 %) and was isolated from seven animals on three farms in three study sites (Adaa, Sululta and Barake districts). *S.* Saintpaul (6, 20 %) was isolated only from a single farm in the Adaa district, *S*. Kentucky (5, 16.7 %), and *S*. Virchow (5, 16.7 %) were isolated from five animals each in two different farms in Addis Ababa, while *S*. Dublin (3, 10 %) and one isolate (1, 3.3 %) of *S*. Livingstone var.14+, *S*. I: 6, 7, 14:-: I,w, *S*. Mikawasima and *S*. Aberdeen were isolated from one animal on five different farms. Two different serotypes were isolated from two farms in the present study (Table 4).

**Table 3:** *Salmonella* serotype distribution and number and percent of intermediate and resistant isolates to antimicrobial agents

Serotype	Number	No. of intermediately resistant and resistant isolates																									
		Amp		Amc		Cf		Cip		Gm		K		Tmp		Te		Su		S		Nitro		Na		N	
		I	R	I	R	I	R	I	R	I	R	I	R	I	R	I	R	I	R	I	R	I	R	I	R	I	R
Aberdeen	1	–	–	–	–	–	–	1	–	–	–	1	–	–	–	1	–	1	–	1	–	–	1	1	–	1	–
Dublin	3	–	–	–		2	–	–	–	–	–	–	–	–	–	–	–	1	–	3	–	–	–	–	–	–	–
I:6,7,14:–:I,w	1	–	1	–	1	–	1	1	–	–	–	–	–	–	–	1	–	–	–	1	–	–	1	1	–	–	–
Kentucky	5	–	5	2	3	–	5	–	5	–	5	4	–	–	1	–	5	–	5	–	5	4	1	–	5	–	2
LivingstoneVar.14+	1	–	–	–	–	–	–	1	–	–	–	–	–	–	–	–	–	–	–	–	–	–	–	–	1	–	–
Mikawasima	1	–	–	–	–	–	–	–	–	–	–	–	–	–	–	–	–	1	–	–		1	–	–	–	–	–
Saintpaul	6	1	–	–	–	1	–	–	–	–	–	4	–	–	–	4	–	3	2	5	1	2	3	–	–	3	–
Typhimurium	7	–	–	–		2	–	–	–	–	–	3	–	–	–	4	–	3	–	7	–	1	1	1	–	1	–
Virchow	5	–	3	3	–	–	3	1	–	1	1	1	–	–	–	–	1	1	1	1	2	1	3	–	2	–	–
Total	30	1	9	5	4	5	9	4	5	1	6	13	–	–	–	10	6	10	8	18	8	9	10	3	8	5	2
% R		3.3	30	16. 7	13.3	16. 7	30	13.3	16.7	3.3	20	43.3	–	–	3.3	33.3	20	33.3	26.7	60	26.7	30	33.3	10	26.7	16.7	6.7
% (I + R)		33.3		30		46.7		30		23.3		43.3		3.3		53.3		60		86.7		63.3		36.7		23.3	

Since all isolates were susceptible to Amikacin, Chloramphenicol, Cefoxitin, Ceftriaxone and Sulfamethoxazole + Trimethoprim, they were not included in the table

*Amp* Ampicillin, *Amc* Amoxicillin and clavulanic acid, Cf Cephalothin, *Cip* Ciprofloxacin, *Gm* Gentamicin, *K* Kanamycin, *Tmp* Trimethoprim, *Te* Tetracycline, *Su* Sulfisoxazole, *S* Streptomycin, *Nitro* Nitrofurantoin, *Na* Nalidixic acid, *N* Neomycin, *I* Intermediate, *R* Resistant

**Table 4:** *Salmonella* serotypes isolated from dairy cattle in various study sites and their antimicrobial resistance pattern

Number	Study site	Farm Code	Serotype	R-pattern	
				Intermediate	Resistant
1	Adaa	DZC −03	Aberdeen	CipKTeSuSNaN	NitroS
2	Adaa	DZC −03	Saintpaul	NitroSuS	–
3	Adaa	DZC −03	Saintpaul	TeSuS	Nitro
4	Adaa	DZC −03	Saintpaul	AmpCfKTeSNaN	SuNitro
5	Adaa	DZC −03	Saintpaul	KTeN	SuSNitro
6	Adaa	DZC −03	Saintpaul	KS	*–*
7	Adaa	DZC −03	Saintpaul	NitroKTeSuSN	
8	Adaa	DZC-06	Typhimurium var. copehagen PT 193	CfKS	*–*
9	Adaa	DZC-06	Typhimurium var. copehagen PT 193	CfTeSuS	*–*
10	Adaa	DZC-06	Typhimurium var. copehagen PT U285	KTeSuSNitroNaN	*–*
11	Adaa	DZC-06	Typhimurium var. copehagen PT193	TeS	*–*
12	Addis Ababa	AAC-25	I:6,7,14:-:I,w	CipTeSNa	AmpAmcCfNitro
13	Addis Ababa	AAC-38	Kentucky	KNitro	AmpAmcCfCipGmTeSuSNa
14	Addis Ababa	AAC-25	Kentucky	Nitro	AmpAmcCfCipGmTeSuSNa
15	Addis Ababa	AAC-38	Kentucky	KNitro	AmpAmcCfCipGmTeSuSNa
16	Addis Ababa	AAC-38	Kentucky	AmcKNitro	AmpCfCipGmTeSuSNaN
17	Addis Ababa	AAC-38	Kentucky	AmcK	AmpCfCipGmTmpTeSuSNitroNaN
18	Addis Ababa	AAC-25	Livingstone Var.14+	Cip	Na
19	Addis Ababa	AAC-09	Mikawasima	SuNitro	*–*
20	Addis Ababa	AAC-23	Virchow	Amc	AmpCf
21	Addis Ababa	AAC-23	Virchow	AmcK	AmpCfCipGmTeSuSNitroNa
22	Addis Ababa	AAC-23	Virchow	AmcSu	AmpCfSNitroNa
23	Addis Ababa	AAC-23	Virchow	GmSNitro	*–*
24	Addis Ababa	AAC-24	Virchow	–	Nitro
25	Barake	BAR- 18	Typhimurium PT Atypical	KSuS	Nitro
26	Barake	BAR- 18	Typhimurium PT 67	S	*–*
27	Sebeta	SC-04	Dublin	Cf,S	*–*
28	Sebeta	SC-04	Dublin	CfSuS	*–*
29	Sebeta	SC-04	Dublin	S	–
30	Sululta	Suc-07	Typhimurium var. copehagen PT Atypical	TeS	*–*

*PT* Phagetype, *Amp* Ampicillin, *Amc* Amoxicillin and clavulanic acid, Cf Cephalothin, *Cip* Ciprofloxacin, *Gm* Gentamicin, *K* Kanamycin, *Tmp* Trimethoprim, *Te* Tetracycline, *Su* Sulfisoxazole, *S* Streptomycin, *Nitro* Nitrofurantoin, *Na* Nalidixic acid, *N* Neomycin

### Antimicrobial resistance

The common antimicrobials used in the farms were oxytetracycline, penicillin + streptomycin, and sulfonamide in 94.6, 81.8 and 13.6 % of the farms, respectively. Resistance patterns of the isolates are shown in Table 4. All isolates were resistant to at least one of the 18 antimicrobials tested. Twenty-six (86.7 %), 19 (63.3 %), 18 (60 %), 16 (53.3 %) of the isolates were resistant to streptomycin, nitrofurantoin, sulfisoxazole and tetracycline, respectively. Resistance to two or more antimicrobials was recorded in 90 % of the isolates, while resistance to 3 or more antimicrobials was detected in 21 (70 %) of the isolates. MDR to 7 or more antimicrobials were detected in 11 (36.7 %) of the isolates. The five *S.* Kentucky isolates were resistant to 10–13 antimicrobials (Table 4). One isolate (*S.* Kentucky) from a farm in Addis Ababa was resistant to 13 out of 18 antimicrobials tested. All isolates were susceptible to amikacin, chloramphenicol, cefoxitin, ceftriaxone and sulfamethoxazole + trimethoprim.

There was a statistically significant difference in the rate of occurrence of MDR between isolates obtained from dairy farms in Addis Ababa and outside of the city limits of Addis Ababa. The mean ± standard error of mean (SEM) number of antimicrobials to which isolates obtained from Addis Ababa were resistant was 7.23 ± 1.32, while isolates obtained outside of Addis Ababa were resistant to 4 ± 0.62 antimicrobials (*p* = 0.01). Resistance to first line antimicrobial agents in human medicine for treatment of *Salmonella* like beta-lactam and quinolones was also more common in isolates obtained from Addis Ababa. The extent of MDR varied with the serotype, as the overall MDR was more common in *S.* Kentucky, *S.* Virchow and *S.* Saintpaul compared to strains from other serotypes. Interestingly, all of the 5 Kentucky strains were resistant to nalidixic acid and ciprofloxacin (Table 4).

## Discussion

Food animals are the primary sources for transmitting non-typhoidal *Salmonella* to humans [27]. Outbreaks of salmonellosis in humans has been linked to improperly pasteurized dairy products, undercooked beef, water runoff from farms, and direct animal or fecal contact [28]. In the current study, farm level prevalence of *Salmonella* was 7.6 % and individual animal level prevalence was 2.3 %, which is much lower than the previous studies in the USA, where 31 % of dairy farms had at least one cow shedding *Salmonella* in feces and 7.3 % of individual animals were shedding [29]. It is also much lower than a previous study conducted in Addis Ababa [15] that reported farm level fecal prevalence of 47.8 % and individual animal level prevalence of 7.7 %. A study on slaughtered cattle in Addis Ababa recovered *Salmonella* from 7.1 % of apparently healthy animals [30]. Recent study in Jordan showed 23 % and 4 % of herd level and individual animal level prevalence of *Salmonella* in dairy farms, respectively, which is also higher than our finding [31]. This difference could be due to differences in the *Salmonella* isolation protocol employed in each study, seasonal variation in *Salmonella* shedding of animals as well as other factors such as herd size and age composition [28, 29]. Most of the farms in the current study had small herd size. Moreover, animals of all age groups in the farm were sampled in the current study unlike the other two studies [15, 29] which involved only lactating cows.

This study also showed that *Salmonella* shedding was common in farms that keep animals completely indoors while none was detected in those that occasionally graze outside or are totally outdoors. Similarly, higher prevalence of *Salmonella* was reported in swine kept indoors than those kept outdoors [32]. This probably is due to free cycling of *Salmonella* between animals in a limited host environment once the pathogen gets access to the farm in animals kept indoors. The fact that the use of processed feed is more common in animals kept indoors than those kept outdoors might also suggest the possibility of indoor kept animals being infected with *Salmonella* from contaminated animal feed. A previous study has also shown livestock waste generated by animals consuming a diet principally composed of grass were less likely to harbor *Salmonella* spp. [33].

In this study, the larger the herd size, the higher the probability of having *Salmonella* positive animals in the farm, which is in agreement with previous reports [34–37]. This could be due to overcrowding of animals in the larger herds, especially those housed indoors, increasing animal to animal contact which enhances transmission of pathogens within the herd. Moreover, the larger the number of animals in the herd, the higher the probability of having a few weak and stressed animals, which increases the likelihood of continuous shedding of *Salmonella* from these cattle. Asymptomatic carrier cattle have been reported to shed *Salmonella* for up to 18 months [38]. Additionally, in the absence of mechanized feeding and milking systems in Ethiopia, several animal attendants are involved in daily activities of the large farms with the possibility of serving as a source of dissemination among individual animals in a farm. Contrary to the above findings, another study has reported that there is no association of herd size and *Salmonella* shedding [29].

The strong association of individual animal and herd level prevalence of *Salmonella* with detection of diarrhea in one or more animals suggests that *Salmonella* is one of the causes of diarrhea in dairy cattle in the study population. Detection of more *Salmonella* from diarrheic as well as non-diarrheic cattle in farms with one or more diarrheic animal in the herd might be due to the presence of carrier animals shedding *Salmonella* to other animals without showing clinical manifestations post infection or after recovery from clinical salmonellosis [39].

The higher *Salmonella* recovery rate in young animals in the current study is presumably due to the lack of an adequate adaptive immune response in the young calves compared to adult animals. Also, co-infection with multiple enteric pathogens is common in calves and may compromise their immune system. In addition, relative lack of protective microflora in calves may also predispose them to pathogenic organisms [37]. A previous study has similarly reported an inverse relationship of calf age and the prevalence of *Salmonella* [40].

The dominant serotypes isolated from dairy cattle in the current study, *S.* Typhimurium*, S.* Saintpaul, *S.* Virchow and *S.* Kentucky, are among the common causes of non-typhoidal salmonellosis in humans [41–43]. There is no previous report in Ethiopia showing serotype distribution of *Salmonella* in dairy cattle. The top three serotypes in slaughtered cattle in Addis Ababa were *S.* Mishmarhaemek, *S.* Typhimurium and *S.* Enteritidis [30]. In another study conducted in north Ethiopia in slaughtered cattle, *S.* Typhimurium and *S.* Newport were the two dominant serotypes recovered [44].

The observed high resistance to streptomycin and tetracycline is not surprising since these antimicrobials are commonly used in most of the farms for management of bacterial infections. Similar high resistance rates were reported to streptomycin (77 %) and tetracycline (65.5 %) in *Salmonella* isolates obtained from different food animals from Ethiopia [45]. Another study also reported 75 and 46.9 % resistance to streptomycin and tetracycline in *Salmonella* isolated from different food items and personnel in Addis Ababa [46]. Though nitrofurantoin and sulfonamide were less commonly used in the farms during the study period, large proportion of isolates were resistant to these agents. This is probably due to the fact that these antimicrobials had been used in the animal health sector for a long time in the country and *Salmonella* had already developed resistance. A previous study conducted in Addis Ababa [15] showed 83 % of *Salmonella* isolates from dairy farms to be resistant to 2 or more antimicrobials out of 10 antimicrobials tested. Unlike the previous study [15] that reported 100 % resistance to ampicillin, in the current study, only 33.3 % of the isolates exhibited resistance to ampicillin. However, most of the isolates obtained from farms in Addis Ababa were resistant to ampicillin.

Resistance to ciprofloxacin was not reported in the previous study [15], but in the current study, 30 % of the isolates were resistant to ciprofloxacin. Despite detection of resistance to ciprofloxacin and nalidixic acid in the current study, from our interview with farm owners during sampling, none of the farms was using ciprofloxacin or other quinolone antimicrobials to treat their dairy cattle, and use of quinolones is not a regular practice in veterinary medicine in Ethiopia. However, a similar high percentage of resistance to ciprofloxacin was observed in *S*. Kentucky isolates carrying *Salmonella* Genomic Island K (SGI1-K) collected during 2000–2008 from meat of swine, cattle and poultry [45]. Also, recently isolated *S*. Kentucky strains from diarrheic patients in Addis Ababa were resistant to several antimicrobials including ciprofloxacin [47]. MDR *S*. Kentucky belonging to a single clone resistant to quinolones and carrying SGI1-K has been reported from European travelers returning from different African and Asian countries [48]. This occurrence of MDR *S*. Kentucky in both humans and animals in the region might be due to this specific clone widely circulating in Africa.

The high MDR in *Salmonella* from dairy cattle is alarming. The incidence of MDR in *Salmonella* has increased in the last few decades globally [49]. Infection of humans with MDR strains of *Salmonella* has been reported to be associated with increased burden of morbidity, extended hospitalization, increased risk of invasive illness and increased mortality, compared to those infected with susceptible strains [50–52]. In fact, the increase in MDR observed in *Salmonella* isolates from dairy farms in Addis Ababa compared to those out of Addis Ababa could be due to greater availability of antimicrobial agents and extensive use of antimicrobials in both animals and humans, fostered by a highly populated city where animals and humans live in close proximity.

## Conclusion

The occurrence of MDR *Salmonella* serotypes that commonly cause human salmonellosis in dairy herds residing in close proximity with human populations warrants the need for strict biosecurity and intervention strategies to control these *Salmonella* isolates in dairy farms and to protect human and animal health. Paramount is the resistance to ciprofloxacin, which is of great concern as this antimicrobial is among the last options for treatment of complicated non-typhoidal salmonellosis in humans.

## Abbreviations

BPW: buffered peptone water
MDR: multi-drug resistance
PT: phage type
RVB: Rappaport-Vassiliadis broth
SEM: standard error of the mean
TTB: tetrathionate broth
WHO: World Health Organization
